# WT1-AS promotes cell apoptosis in hepatocellular carcinoma through down-regulating of WT1

**DOI:** 10.1186/s13046-015-0233-7

**Published:** 2015-10-13

**Authors:** Long Lv, Gong Chen, Jianping Zhou, Jun Li, Jianping Gong

**Affiliations:** Department of General Surgery, People’s Hospital of Gaochun, No. 9 Chunzhong Road, Gaochun, Nanjing, 211300 Jiangsu Province China; Department of General Surgery, Yixing People’s Hospital, the Affiliated Hospital of Jiangsu University, Yixing, 214200 Jiangsu Province China; Department of General Surgery, Nanjing Jiangning Hospital, the Affiliated Jiangning Hospital of Nanjing Medical University, Nanjing, 211100 Jiangsu Province P.R. China; Department of General Surgery, Jiangsu Cancer Hospital Affiliated to Nanjing Medical University, Nanjing, 210009 China

**Keywords:** Hepatocellular carcinoma, Promoter, JAK2/STAT3, Chemotherapy

## Abstract

**Background:**

The antisense of the tumor suppressor gene WT1 (WT1-AS) is a long non-coding RNA. The role of WT1-AS in the development of hepatocellular carcinoma (HCC) has not yet been elucidated.

**Methods:**

Quantitative real-time PCR and western blot analyses were used to measure levels of WT1-AS and its related genes in tumor and corresponding adjacent tumor tissues of HCC patients. The effect on HCC cell proliferation and apoptosis was assessed by EdU incorporation assays and PI-Annexin-V staining, respectively. ShRNA and dual-luciferase assays were used to investigate the regulatory relationship between WT1-AS and WT1 in cell lines.

**Results:**

WT1-AS expression correlated negatively with WT1 expression in HCC tumor tissue. Kaplan-Meier curve analysis revealed that WT1-AS expression is a reliable indicator of HCC prognosis. The downregulation of WT1 expression by WT1-AS promoted cell apoptosis by suppressing the JAK/STAT3 signaling pathway. Bioinformatics analysis showed that WT1-AS downregulates WT1 by binding to the TATA region of the WT1 promotor. WT1-AS was also able to reverse WT1-mediated resistance to Dox based chemotherapy in HCC cells.

**Conclusions:**

WT1-AS downregulates WT1 expression in HCC tumors and promotes apoptosis by binding to the promoter region of WT1. Our findings suggest that WT1-AS may function as a tumor suppressor in HCC by reversing the oncogenic effects of WT1.

**Electronic supplementary material:**

The online version of this article (doi:10.1186/s13046-015-0233-7) contains supplementary material, which is available to authorized users.

## Introduction

Hepatocellular carcinoma (HCC) is the sixth most common malignant tumor and is the third leading cause of cancer-related death worldwide [1]. There are several approaches to treating HCC, including surgical resection, liver transplantation, transcatheter arterial chemoembolization and local radiofrequency ablation, but none have improved the overall long-term survival of patients. This is largely attributed to the high malignancy and efficient intrahepatic invasion of HCC tumors, which leads to frequent reoccurrence after treatment [2, 3]. Novel biomarkers or HCC treatments are therefore in considerable demand.

Wilms’ tumor 1 gene (WT1) is located on chromosome 11p13 [4] and regulates several genes including PDGF-A chain, IGF-II, IGF-IR, c-myc and bcl-2 [5]. WT1 regulates transcription, RNA metabolism, translation and both oncogenic and tumor suppressor functions [6]. Researchers have found that the over-expression of WT1 acted as an oncogene in the pathogenesis of neoplasm [7]. WT1 overexpression has now been reported in various tumors and is predictive of a poor prognosis in breast cancer [8]. A similar effect for WT1 has also been reported in HCC; WT1 overexpression has been associated with poor prognosis, enhanced tumor progression and resistance to chemotherapy [9]. Moreover, overexpression of WT1 have been investigated in human HCC, related to poor prognosis of post-surgery patients and contributed to tumor progression and resistance to chemotherapy [10, 11], to explore a novel therapeutic approach for HCC, we investigated the causes of WT1 upregulation in HCC tumor tissue.

The long non-coding RNAs (lncRNAs) are important for predicting survival and metastasis in liver cancer [12]. LncRNAs regulate the progression of liver disease by a variety of mechanisms including DNA imprinting, X inactivation, DNA demethylation, gene transcription and RNA synthesis [13]. Furthermore, several reports have demonstrated that lncRNAs are subjected to epigenetic modifications, including methylation, ubiquitination and miRNA-induced regulation [14].

## Materials and methods

### Patient samples

Study data were obtained from 90 patients who presented between September 2012 and October 2013 at The First Affiliated Hospital of Nanjing Medical University (Nanjing, China). Informed consent for tissue analysis was obtained prior to surgery; the study was approved by our Institutional Ethics Committee. All research was performed in compliance with government policies and the Helsinki Declaration. Experiments were undertaken with the understanding and written consent of each subject. The clinical information was analyzed in Table 1.

**Table 1:** Correlation between expression level of WT1-AS and clinical characteristic of patients

	WT1-AS		
Character	Low	High	*P* value
	45	45	
Age			
< 60	30	28	>0.05
≥ 60	15	17	
Gender			>0.05
Male	38	37	
Female	7	8	
Tumor Size (cm)			<0.05
≤ 5 cm	17	27	
> 5 cm	28	18	
Tumor Capsular			>0.05
Incomplete	23	22	
Complete	22	23	
TNM stage (I:II:III)	19:16:10	20:15:10	>0.05

### Quantitative RT-PCR

Quantitative real time polymerase chain reaction (qRT-PCR) was performed to determine the expression levels of WT1-AS of all related genes. Total RNA was obtained from tissues using TRIzol reagent as described by the manufacturer (Invitrogen Life Technologies Co, CA, USA). For mRNA detection, total RNAs (500 ng) were reverse transcribed using the reverse transcription kit (Takara, Tokyo, Japan) at 37 °C for 15 min and 85 °C for 30 s. QRT-PCR was performed using ABI Prism 7900HT (Applied Biosystems, CA, USA). β-actin was used as an internal control. PCR was performed for 5 s at 95 °C and for 30 s at 60 °C for 40 cycles. The detailed sequence information was presented in additional file 1 online.

### Protein analysis

For Western-blots, total proteins were extracted from tissues or cultured cells using RIPA buffer containing protease inhibitors cOmplete, ULTRA, Mini, EDTA-free, EASYpack (Roche, Basel, Switzerland), while the membrane proteins were extracted from tissues by Mem-PER Eukaryotic Membrane Protein Extraction Reagent Kit (Thermo Scientific, Rockford, USA). Protein concentrations were determined by the BCA method. Equal amount of proteins (100ug) were separated with 7.5 %/12.5 % sodium dodecyl sulphate polyacrylamide gel electrophoresis (SDS-PAGE) and transferred to PVDF membrane. Membrane was blocked using 5 % skimmed milk and incubated with respective antibodies. Primary polyclonal antibodies were purchased from Santa Cruz Biotechnology (CA, USA) and Abcam (CA, USA) including WT1 (ab96792), p-STAT3 (ab30647), STAT3 (ab68153), p-MAPK (ab63378), MAPK (ab197348), GAPDH (sc-48166), β-actin (sc-47778), Caspase 3 (ab2302), MDR1 (sc-13131). The secondary antibodies were anti-rabbit or anti-mouse HRP-linked were purchased from Santa Cruz Biotechnology (CA, USA). The blots were developed using ECL reagent (Millpore, MASS, USA). Equal amount of protein loading in each lane was confirmed using GAPDH antibody. All experiments were repeated at least three times.

### Cell culture and reagents

Human HCC cell lines were obtained from American Type Culture Collection (ATCC, Manassas VA, USA), which were cultured in complete growth medium DMEM (Hyclone, UT, USA), supplemented with 10 % fetal bovine serum (10 % FBS), 100 U/mL penicillin, and 100 μg/mL streptomycin at 37 °C, 5 % CO_2_ Cells were cultured to about 50 % confluence and transfection was carried out using Lipofectamine 2000 (Invitrogen Corp, CA, USA).

### Cell apoptosis and proliferation assay

For the apoptosis analysis, cells were washed in PBS, and then processed with Annexin V-FITC Apoptosis Detection Kit (BD Biopharmingen, NJ, and USA) for 15 min in the dark. All experiments were analyzed by BD Biasciences FACS Calibur Flow Cytometry (BD Biasciences, NJ, and USA). The tests were repeated for three times with triplicate per experiment. Cell proliferation was assayed by using EDU (5-ethynyl-2′-deoxyuridine). (Roche, Basel, Switzerland) as described previously [15].

### Fluorescent In Situ Hybridization (FISH)

Cells were briefly rinsed in PBS and fixed in 4 % formaldehyde in PBS (pH 7.4) for 15 min at room temperature. Then the cells were permeabilized in PBS containing 0.5 % Triton X-100 on ice for 10 min; washed with PBS and rinsed once in 2 × SSC prior to hybridization. Using an anti-WT1-AS oligodeoxynucleotide probe which was conjugated with Alexa Fluor 488 (Invitrogen, Carlsbad, CA), hybridization was performed in hybridization solution (probe dilution 1:1250) (Boster, China) for 16 h at 50 °C in a moist chamber. For FISH, cells were washed for 30 min in 25 % deionized formamide/2 × SSC at 50 °C and then at 50 °C, 30 min in 2 × SSC.

### Dual-luciferase reporter assay

The promoter sequence of WT1 predicted to interact with WT1-AS or a mutated sequence with the predicted target sites were inserted into the KpnI and SacI sites of pGL3 promoter vector (Genscript, Nanjing, China). These constructs were named pGL3-WT1, and pGL3-WT1 mut. For reporter assay, cells were plated onto 24-well plates and transfected with 100 ng of pGL3-WT1, pGL3-WT1-mut, respectively, using Lipofectamine 2000 (Invitrogen Corp, CA, and USA). A Renilla luciferase vector pRL-SV40 (5 ng) was also co-transfected to normalize the differences in transfection efficiency. After transfection for 48 h, cells were harvested and assayed with the Dual-Luciferase Reporter Assay System (Promega, Madison, WI) according to the manufacturer’s instructions. Transfection was repeated three times in triplicate.

### Data analysis

All experiments were independently repeated at least triplicate. Data were expressed as mean ± SD. Chi-square tests was used to evaluate statistical differences in demographic and clinical characteristics in HCC patients. Differences between two independent groups including clinical samples and in vitro study were tested with the student *t* test. Pearson correlation was applied to analyze the correlation between WT1 and WT1-AS. All statistical analyses were carried out using SPSS version 18.0 and presented with Graphpad prism software. Kaplan-Meier survival curves were plotted and log rank test was done. The significance of various variables for survival was analyzed by Cox proportional hazards model in a multivariate analysis. The results were considered to be statistically significant at *P* < 0.05.

## Results

### WT1-AS expression correlated negatively with WT1 expression in HCC tumors

**Fig. 1:**
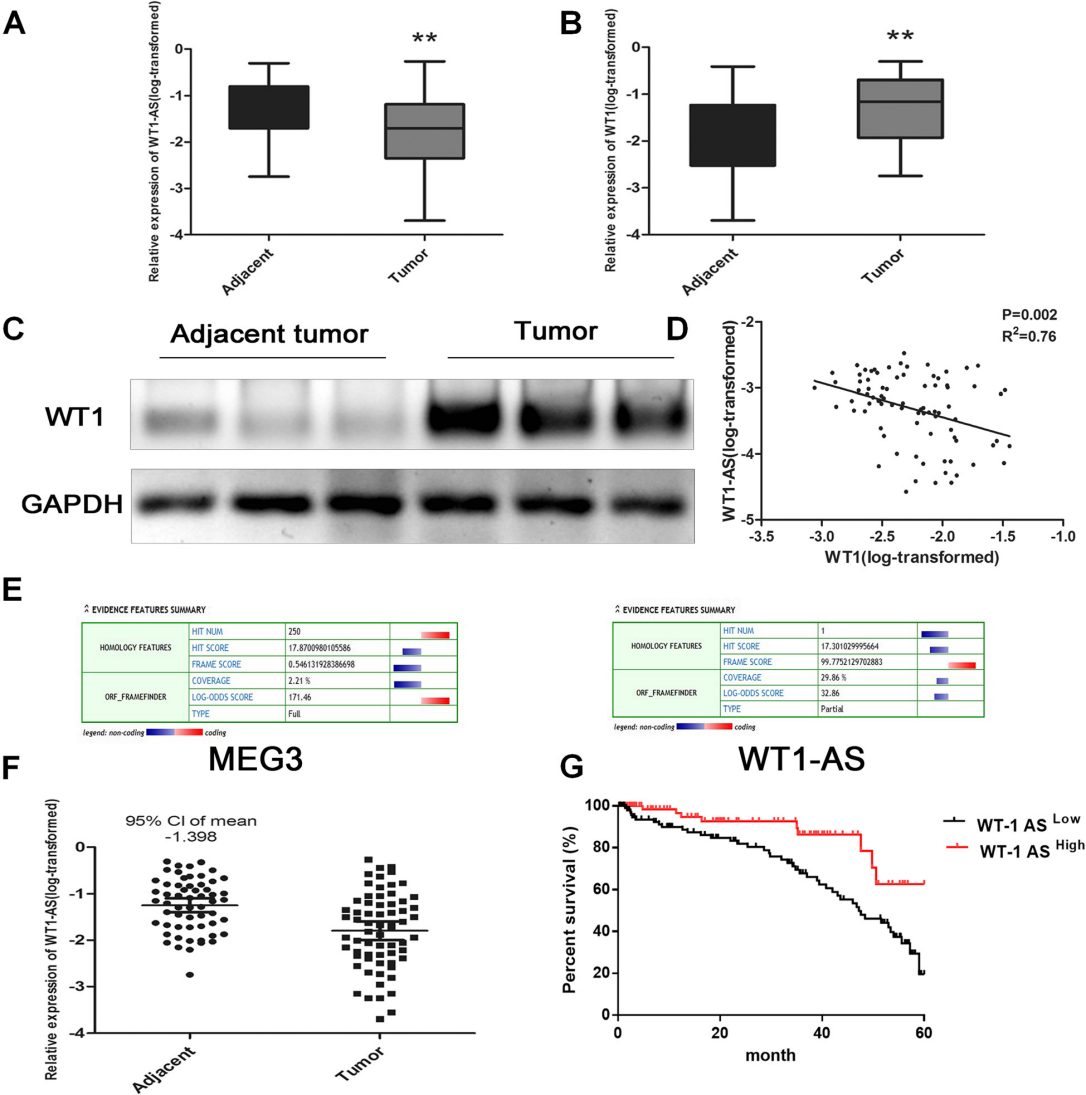
WT1-AS was down-regulated with a high correlation with WT1 in HCC acting as an ideal indicator for good prognosis of human HCC. **a** Down-regulated level of WT1-AS in HCC tissues compared with the corresponding adjacent tissues. **b** Up-regulated level of WT1 mRNA in HCC. **c** Protein expression level of WT1 in HCC tissues. **d** Data were analyzed using the Pearson correlation analysis with natural log transformed expression levels. The result showed that there was an inverse correlation between WT1 and WT1-AS expression levels (*P* = 0.002, R^2^ = 0.76). **e** Coding Protein Calculator (http://www.lncipedia.org/) was employed to examine the protein coding ability of WT1-AS. MEG3 was regarded as a positive control. **f** Expression of WT1-AS in human HCC and adjacent normal tissues. The threshold discriminating normal from elevated systemic levels was obtained using 95 % CI in the healthy controls. **g** The Kaplan–Meier curve for the overall survival of patients was presented by using WT1-AS expression

In order to investigate whether changes in WT1-AS expression are associated with HCC, we quantified the expression of WT1-AS in tumor tissues and the corresponding adjacent tissues. WT1-AS expression was significantly reduced in tumor tissue compared to normal tissues (Fig. 1a), indicating that WT1-AS is involved in HCC. We also confirmed the up-regulation of WT1 expression in HCC tumors (Fig. 1b, c).

The correlation of WT1-AS and WT1 expression and the ability of WT1-AS to regulate WT1 have not been investigated so far. To address this, we analyzed the correlation of WT1-AS and WT1 expression using Pearson correlation analysis. We confirmed an inverse correlation between WT1-AS and WT1 expression (Fig. 1d).

The Coding Protein Calculator (http://cpc.cbi.pku.edu.cn/) was used to examine the protein coding ability of WT1-AS. Comping with MEG3, WT1-AS was shown to be a non-coding RNA (Fig. 1e).

### WT1-AS is a reliable predictor of HCC prognosis

The expression of WT1-AS in HCC and adjacent normal liver tissues was re-evaluated. Evaluation of the 95 % CI in the adjacent normal tissues group indicated a threshold of −0.1398 for discriminating normal tissue from tumor tissue. Therefore, we divided our 65 patients into two groups; the WT1-AS^high^ group (34 cases) and the WT1-AS^low^ group (31 cases). The 5-year survival rate in the WT1-AS^high^ group was significantly higher than the WT1-AS^low^ group (*P* = 0.0018, 95 % CI). WT1-AS might be a reliable indicator for the prognosis of HCC (Fig. 1f).

### WT1-AS suppresses proliferation and promotes apoptosis in human HCC cells

**Fig. 2:**
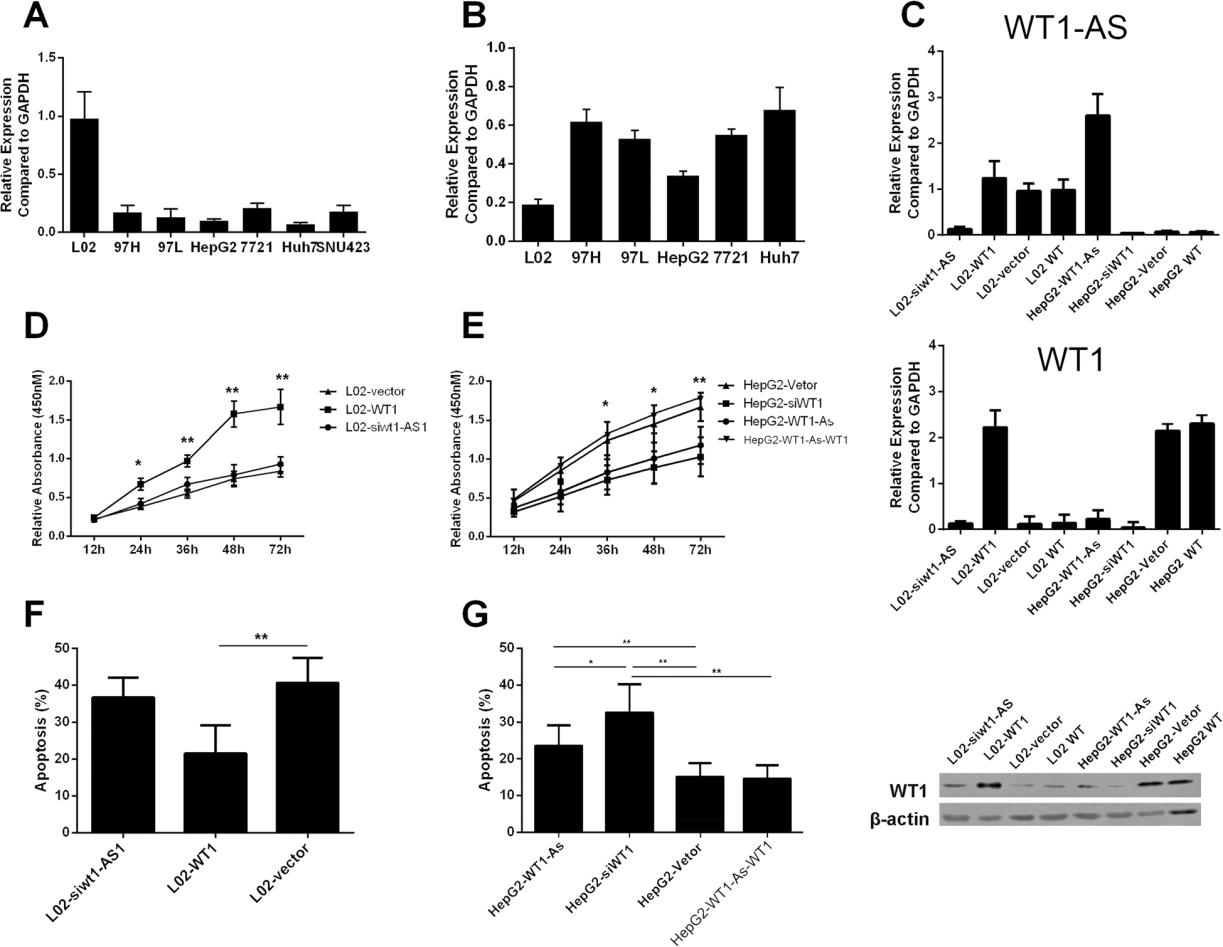
Apoptosis promoting and proliferation inhibiting effect of WT1-AS by targeting WT1. **a** and **b** Expression of WT1-As and WT1 determined in various human HCC cell lines indicated in the figure. **c** Detection of WT1-AS and WT1 expression by real-time PCR after transfection (*Upper panel*). WT1 expression by western-blot in L02 and HepG2 cells treated with WT1-AS (*lower panel*). **d** and **e** Proliferation curve exampled by EdU assay for L02 and HepG2 cells treated with various conditions. **f** and **g** Cell apoptosis determined detected by PI-Annexin V staining assay for L02 and HepG2 cell treated with various condition indicated in the figure. All data presented as mean ± SD, *, *P* < 0.05, **, *P* < 0.01 by unpaired Student’s *t* test

WT1 and WT1-AS expression was measured in the human HCC cell lines 97H, 97 L, HepG2, SMCC-7721, SNU-423 and Huh7. As a control, expression was also measured in the normal human hepatocyte cell line L02. WT1-AS expression was significantly lower in the HCC cell lines compared to L02 cells (Fig. 2a). In contrast, WT1 expression was significantly higher in the HCC cell lines compared to L02 cells (Fig. 2b). We performed further functional investigations in the HepG2 and L02 cell lines.

We manipulated WT1-AS and WT1 expression in L02 and HepG2 cells by overexpression and shRNA and investigated the reciprocal effect on protein and mRNA expression by western-blot and real-time PCR (Fig. 2c). Downregulation of WT1-AS expression by shRNA in L02 cells did not affect WT1 transcription, whereas overexpression of WT1-AS in HepG2 cells significantly down-regulated the level of WT1 mRNA, suggesting that WT1-AS might downregulate WT1 expression in HCC through a direct interaction rather than by blocking transcription.

Overexpression of WT1 significantly increased the proliferation of L02 cells (L02-WT1), but the downregulation of WT1-AS had no influence on the proliferation of L02 cells (L02-shT1-AS). In contrast, the proliferation of HepG2 cells was significantly decreased by the downregulation of WT1 (HepG2 shWT1) or the overexpression of WT1-AS (HepG2 WT1-AS). Overexpressing WT1 in HepG2 WT1-AS cells was sufficient to reverse the effect of WT1-AS on HepG2 cell proliferation (Fig. 2d, e).

We investigated the effect of WT1 and WT1-AS on cell apoptosis using a H_2_O_2_ induced model (Fig. 2f, g). The rate of apoptosis decreased significantly in L02 cells when WT1 was overexpressed. Interestingly, WT-AS1 knockdown had no effect on apoptosis in L02 cells. In HepG2 cells, apoptosis increased significantly when WT1 was knocked down or when WT1-AS was overexpressed. The greater increase in apoptosis was induced by WT1 knock-down and was rescued by re-transfection of WT1.

### WT1-AS controls WT1 expression through a reciprocal feedback loop

**Fig. 3:**
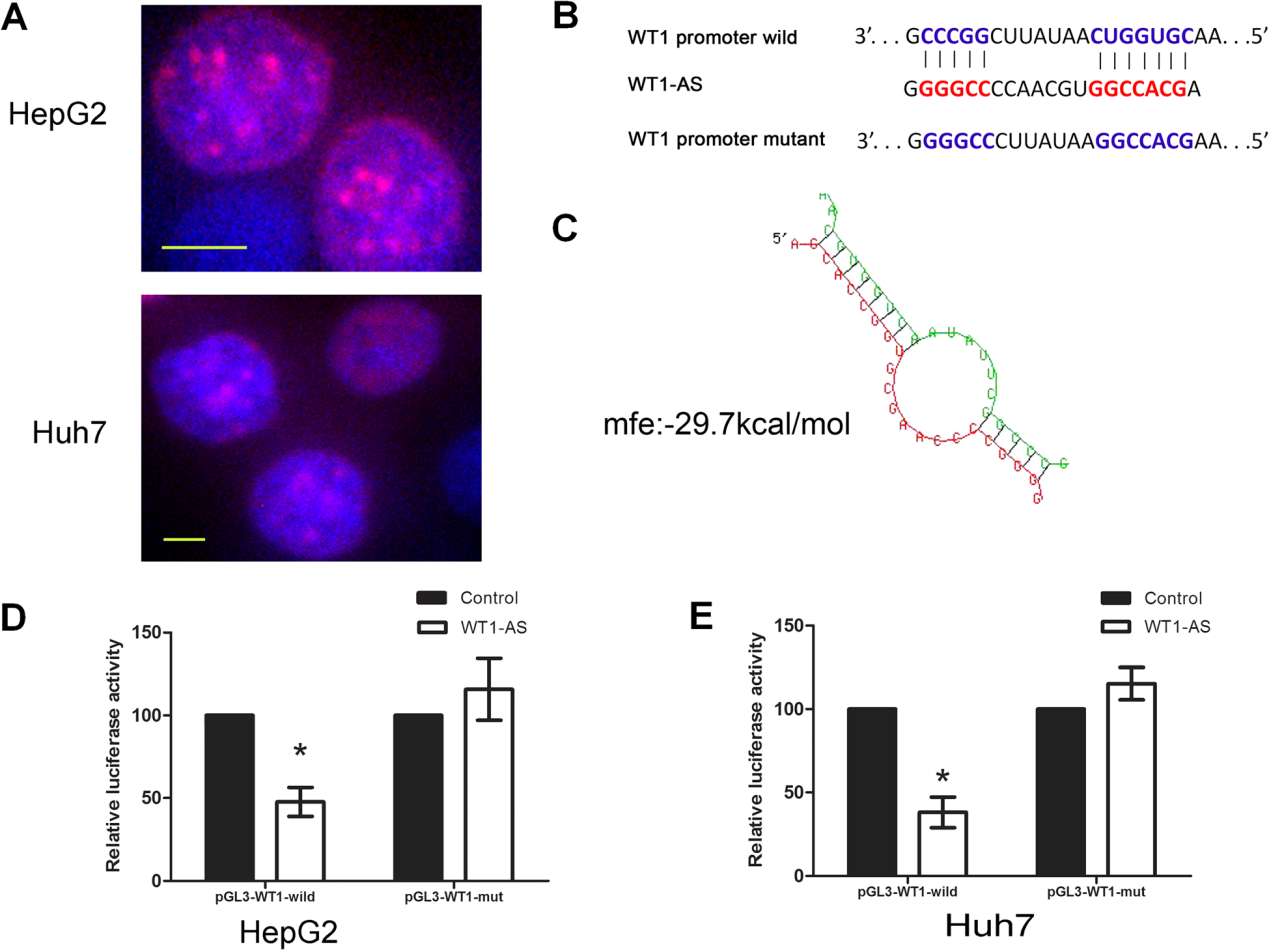
WT1-AS reciprocally control WT1 expression through a feedback loop. **a** Subcellular localization investigation by FISH indicated that the transcript for WT1-AS was located mainly in the nucleus of Huh7 and HepG2 cell lines, the upper panel indicated Huh7 while the down panel indicated HepG2. DAPI was used as the control for labeling the nucleus. **b** Sequences of the binding site for WT1-AS in the promoter region of WT1 and the WT1 promoter and a mutant sequence were cloned for further experiments. Wild type presented in blue while mutant type in red. **c** Bioinformatics predicted the binding site between the WT1-AS with WT1. **d**, **e** Cells were co-transfected with WT1-AS plasmid or control, Renilla luciferase vector pRL-SV40 and WT1 promoter (wild type and mutant type). Both firefly and Renilla luciferase activities were measured in the same sample. Firefly luciferase signals were normalized with Renilla luciferase signals. Cells treated with controls plasmid were normalized to 100 %. Panel **d** presented HepG2 cell line while panel **e** presented Huh7. All data presented as mean ± SD, * indicated *P* < 0.05 by unpaired Student’s *t* test

We used a bioinformatics approach to further explore the relationship between WT1-AS and WT1. FISH assays were performed to investigate the subcellular localization of WT1-AS and WT1 in HCC cell lines. The transcript of WT1-AS was located primarily in the nucleus of Huh7 and HepG2 cells (Fig. 3a). While analyzing a 2-kb region upstream of the transcription start site of WT1 using the UCSC genome browser, we observed that WT1-AS may bind to the WT1 TATA region. The WT1-AS binding site sequences in the promoter region of WT1 are presented in Fig. 3b, c. We observed a reduction of wild-type WT1 luciferase activity when WT1-AS was overexpressed in HepG2 and Huh7 cells (*P* < 0.001), but not when the WT1-AS binding sequences were mutated (Fig. 3d, e). These results suggest that WT1-AS binds to a specific promoter region of WT1 to inhibit transcription.

### WT1-AS negatively regulates WT1-mediated resistance to chemotherapy through JAK2/STAT3 and MAPK signaling

**Fig. 4:**
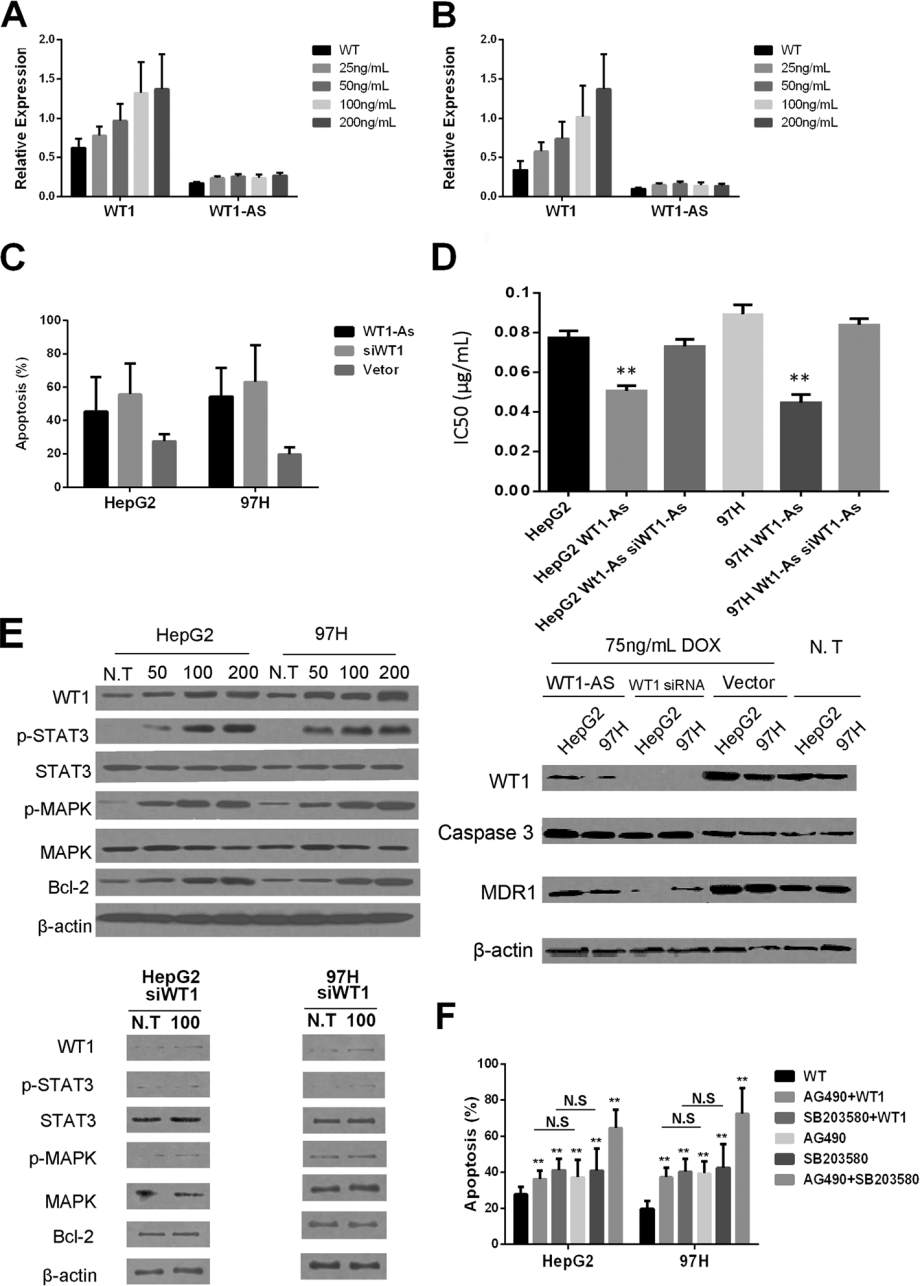
WT1-AS was a negative regulator for chemo-resistance though JAK2/STAT3 and MAPK signaling. **a**, **b** Expression of WT1-AS and WT1 determined by real-time PCR in HepG2 and 97H cells treated with doxorubicin (DOX) at concentration of 25, 50, 100, 200 ng/m. **c** Cell apoptosis determined detected by PI-Annexin V staining assay for 97H and HepG2 treated with either WT1-AS and shRNA targeting WT1. **d** Determination of DOX IC50 on 97H and HepG2 cells treated as indicated in the figure (*upper panel*). Expression of WT1, Caspase 3, and MDR1 in both 97H and HepG2 cells transfected with either WT1-AS and shRNA targeting WT1 and further treated with or without DOX at concentration of 75 ng/mL (*lower panel*). **e** Expression of WT1, p-STAT3, STAT3, p-MAPK, MAPK and Bcl-2 in 97H, HepG2 as well as their siWT1 treated cells treated with various concentration of DOX indicated in the figure. **f** Cell apoptosis determined detected by PI-Annexin V staining assay for 97H and HepG2 cell treated with various conditions indicated in the figure. All data presented as mean ± sd, N.S., No significance, ** indicated *P* < 0.01 by unpaired Student’s *t* test

HCC is commonly treated by chemotherapeutic drugs that inhibit apoptosis [16]. To investigate the association of WT1-AS expression with chemotherapeutic drug resistance, two HCC cell lines with a low expression of WT1-AS and a high expression of WT1 were treated with the commonly used chemotherapeutic drug doxorubicin (DOX). The expression of WT1 increased gradually with the concentration of DOX (25 ng/mL to 200 ng/mL) in 97H and HepG2 cells. The expression of WT1-AS also increased significantly with the treatment of 25 ng/mL DOX to 50 ng/ml DOX, but no further increase of WT1-AS expression was observed with the treatment of higher DOX concentrations (Fig. 4a, b), in agreement with previous findings [10].

The IC_50_ of DOX was increased by WT1-AS overexpression and decreased by WT1-AS knock-down in 97H and HepG2 cell, suggesting that WT1-AS enhances DOX function. Indeed, overexpressing WT1-AS in 97H and HepG2 cells significantly increased the induction of apoptosis by a moderate 75 ng/mL dose of DOX. The same effect was observed when WT1 expression was knocked down in 97H and HepG2 cells. These findings suggested that WT1-AS may regulate DOX-mediated apoptosis by targeting WT1. To explore this, we measured the expression of apoptosis related proteins in HCC cells treated with DOX (Fig. 4c). The expression of the apoptosis marker caspase 3 subunit p17 increased in all groups treated with DOX and this increase was significantly higher when WT1-AS was overexpressed or WT1 was downregulated. We also detected a reduced expression of MDR1, which encodes the multidrug resistance protein P-glycoprotein, when WT1 was downregulated (Fig. 4d).

WT-1 regulates the oncogenicity of leukemia cells by activating JAK/STAT3 and MAPK signaling [17, 18], therefore we explored the role of JAK/STAT3 and MAPK signaling in the WT1-mediated resistance to DOX.

Upregulation of WT1 expression by DOX treatment activated MAPK and STAT3 signaling in HepG2 and 97H cells, as indicated by an increased phosphorylation of STAT3 and MAPK and the increased expression of downstream targets such as BCL-2 (Fig. 4e). Antagonists of MAPK (SB203580) and STAT3 (AG490) signaling were used to confirm the effect of WT1 specifically on these pathways. AG490 and SB203580 treatment significantly increased the apoptosis rate in 97H and HepG2 cells, especially when used in combination. Overexpression of WT1 was not able to rescue apoptosis when either STAT3 or MAPK signaling was blocked (Fig. 4f). Our findings provide evidence that WT1 induces resistance to DOX through JAK2/STAT3 and MAPK signaling.

## Discussion

WT1 encodes a zinc-finger transcription factor and was first described as a tumor suppressor gene in the pediatric kidney cancer, Wilms’ tumor. In normal tissues, WT1 is an important regulator of cell growth and development [19, 20]. It controls embryogenesis and influences the correct formation of many organs and tissues [21, 22]. However, the role of WT1 in cancer is complicated, with conflicting reports as to whether it has a tumor suppressive or oncogenic function [23, 24]. In this study we revealed an oncogenic role for WT1, promoting proliferation and inhibiting apoptosis in HCC cells. WT1 also promoted the resistance of HCC cells to a chemotherapeutic drug, in agreement with previous findings, possibly by activating JAK2/STAT3 and MAPK signaling [10].

We have demonstrated that WT1-AS regulates expression of WT1 in HCC tumors, but not in normal tissue. WT1-AS has previously been implicated in tumorigenesis by interacting with WT1 [25, 26]. therefore our findings imply that WT1-AS can inhibit the growth of HCC tumors, possibly by reversing the oncogenic effect of WT1 on HCC cell proliferation and apoptosis. We have also provided evidence that WT1-AS can attenuate the resistance of HCC cells to a chemotherapeutic drug by influencing the activation of JAK2/STAT3 and MAPK signaling by WT1. WT1-AS appears to regulate WT1 by binding directly to its promoter region.

## Conclusions

We have demonstrated that high WT1-AS expression is indicative of a good prognosis for HCC, possibly by reversing the oncogenic effects of its target WT1.

## Additional file

Additional file 1: Table S1. Sequence information for primer and siRNA. (DOCX 13 kb)

## Abbreviations

GAPDH: Glyceraldehyde 3-phosphate dehydrogenase
HCC: Hepatocellular carcinoma
WT1: Wilms’ tumor 1 gene
lncRNA: Long non-coding RNAs
qRT-PCR: Quantitative real time polymerase chain reaction
SDS-PAGE: Sodium dodecyl sulphate polyacrylamide gel electrophoresis
10 % FBS: 10 % fetal bovine serum
EDU: 5-ethynyl-2′-deoxyuridine
FISH: Fluorescent In Situ Hybridization
DOX: Doxorubicin

## References

[1] Park YY, Kim SB, Han HD, Sohn BH, Kim JH, Liang J et al. TARDBP regulates glycolysis in hepatocellular carcinomaby regulating PFKP through miR-520. Hepatology. 2013. doi:10.1002/hep.26310. 10.1002/hep.26310 PMC3923572 23389994

[2] Bruix J, Llovet JM (2002). Prognostic prediction and treatment strategy in hepatocellular carcinoma. Hepatology.

[3] Schafer DF, Sorrell MF (1999). Hepatocellular carcinoma. Lancet.

[4] Sinha S, Thomas D, Yu L, Gentles A, Jung N, Corces-Zimmerman MR (2014). Mutant WT1 is associated with DNA hypermethylation of PRC2 targets in AML and responds to EZH2 inhibition. Blood.

[5] Xu W, Ji J, Xu Y, Liu Y, Shi L, Lu X et al. MicroRNA-191, by promoting the EMT and increasing CSC-like properties, is involved in neoplastic and metastatic properties of transformed human bronchial epithelial cells. Mol Carcinog. 2014. doi:10.1002/mc.22221. 10.1002/mc.22221 25252218

[6] Kang HJ, Park JH, Chen W, Kang SI, Moroz K, Ladanyi M (2014). EWS-WT1 oncoprotein activates neuronal reprogramming factor ASCL1 and promotes neural differentiation. Cancer Res.

[7] Koido S, Homma S, Okamoto M, Takakura K, Mori M, Yoshizaki S (2014). Treatment with chemotherapy and dendritic cells pulsed with multiple Wilms' tumor 1 (WT1)-specific MHC class I/II-restricted epitopes for pancreatic cancer. Clin Cancer Res.

[8] Krauth MT, Alpermann T, Bacher U, Eder C, Dicker F, Ulke M (2014). WT1 mutations are secondary events in AML, show varying frequencies and impact on prognosis between genetic subgroups. Leukemia.

[9] Berasain C, Herrero JI, Garcia-Trevijano ER, Avila MA, Esteban JI, Mato JM (2003). Expression of Wilms' tumor suppressor in the liver with cirrhosis: relation to hepatocyte nuclear factor 4 and hepatocellular function. Hepatology.

[10] Perugorria MJ, Castillo J, Latasa MU, Goni S, Segura V, Sangro B (2009). Wilms' tumor 1 gene expression in hepatocellular carcinoma promotes cell dedifferentiation and resistance to chemotherapy. Cancer Res.

[11] Sera T, Hiasa Y, Mashiba T, Tokumoto Y, Hirooka M, Konishi I (2008). Wilms' tumour 1 gene expression is increased in hepatocellular carcinoma and associated with poor prognosis. Eur J Cancer.

[12] Wang F, Yuan JH, Wang SB, Yang F, Yuan SX, Ye C (2014). Oncofetal long noncoding RNA PVT1 promotes proliferation and stem cell-like property of hepatocellular carcinoma cells by stabilizing NOP2. Hepatology.

[13] He Y, Meng XM, Huang C, Wu BM, Zhang L, Lv XW (2014). Long noncoding RNAs: Novel insights into hepatocelluar carcinoma. Cancer Lett.

[14] Liu J, Wang DZ (2014). An Epigenetic "LINK(RNA)" to Pathological Cardiac Hypertrophy. Cell Metab.

[15] Tang J, Zhuo H, Zhang X, Jiang R, Ji J, Deng L (2014). A novel biomarker Linc00974 interacting with KRT19 promotes proliferation and metastasis in hepatocellular carcinoma. Cell Death Dis..

[16] Ni Nyoman AD, Luder CG (2013). Apoptosis-like cell death pathways in the unicellular parasite Toxoplasma gondii following treatment with apoptosis inducers and chemotherapeutic agents: a proof-of-concept study. Apoptosis.

[17] Rong Y, Cheng L, Ning H, Zou J, Zhang Y, Xu F (2006). Wilms' tumor 1 and signal transducers and activators of transcription 3 synergistically promote cell proliferation: a possible mechanism in sporadic Wilms' tumor. Cancer Res.

[18] Li X, Li Y, Yuan T, Zhang Q, Jia Y, Li Q (2014). Exogenous expression of WT1 gene influences U937 cell biological behaviors and activates MAPK and JAK-STAT signaling pathways. Leuk Res.

[19] Wagner KD, Cherfils-Vicini J, Hosen N, Hohenstein P, Gilson E, Hastie ND (2014). The Wilms' tumour suppressor Wt1 is a major regulator of tumour angiogenesis and progression. Nat Commun..

[20] Wang Y, Thomas A, Lau C, Rajan A, Zhu Y, Killian JK (2014). Mutations of epigenetic regulatory genes are common in thymic carcinomas. Sci Rep..

[21] Kirschner KM, Braun JF, Jacobi CL, Rudigier LJ, Persson AB, Scholz H (2014). Amine oxidase copper-containing 1 (AOC1) is a downstream target gene of the Wilms tumor protein, WT1, during kidney development. J Biol Chem.

[22] Karki S, Surolia R, Hock TD, Guroji P, Zolak JS, Duggal R (2014). Wilms' tumor 1 (Wt1) regulates pleural mesothelial cell plasticity and transition into myofibroblasts in idiopathic pulmonary fibrosis. FASEB J.

[23] Li X, Wang S, Sitaram RT, Andersson C, Ljungberg B, Li A (2013). Single nucleotide polymorphisms in the Wilms' tumour gene 1 in clear cell renal cell carcinoma. PLoS One.

[24] Iiyama T, Udaka K, Takeda S, Takeuchi T, Adachi YC, Ohtsuki Y (2007). WT1 (Wilms' tumor 1) peptide immunotherapy for renal cell carcinoma. Microbiol Immunol.

[25] Dallosso AR, Hancock AL, Malik S, Salpekar A, King-Underwood L, Pritchard-Jones K (2007). Alternately spliced WT1 antisense transcripts interact with WT1 sense RNA and show epigenetic and splicing defects in cancer. RNA.

[26] Hancock AL, Brown KW, Moorwood K, Moon H, Holmgren C, Mardikar SH (2007). A CTCF-binding silencer regulates the imprinted genes AWT1 and WT1-AS and exhibits sequential epigenetic defects during Wilms' tumourigenesis. Hum Mol Genet.

