# Supplementary material for: WT1-AS promotes cell apoptosis in hepatocellular carcinoma through down-regulating of WT1
Source: J Exp Clin Cancer Res. 2015 Oct 13;34:119. doi: 10.1186/s13046-015-0233-7 (PMC4604772; doi:10.1186/s13046-015-0233-7)
Supplement: Additional file 1: Table S1. — Sequence information for primer and siRNA. (DOCX 13 kb) [file 13046_2015_233_MOESM1_ESM.docx]

**Additional file**

Additional file 1: Table S1. Sequence information for primer and siRNA.

|  | Forward primer（5′--3′） | Reverse primer（5′--3′） |
| --- | --- | --- |
| WT1 | GGGGTAAGGAGTTCAAGGCA | TGCAGCAAGAGGAAGTCCAG |
| WT1-AS | CAGGACTCACAGCCTAGCAC | CTCTGCTACAGATCTCGGCG |
| WT1 siRNA | CCAAATGACATCCCAGCTT |  |
| WT1-AS siRNA | GCTCTCCTGTACCTCCTTT |  |
